# Endemic Severe Fever with Thrombocytopenia Syndrome, Vietnam

**DOI:** 10.3201/eid2505.181463

**Published:** 2019-05

**Authors:** Xuan Chuong Tran, Yeojun Yun, Le Van An, So-Hee Kim, Nguyen T. Phuong Thao, Phan Kim C. Man, Jeong Rae Yoo, Sang Taek Heo, Nam-Hyuk Cho, Keun Hwa Lee

**Affiliations:** The Hue University Hospital and Hue University of Medicine and Pharmacy, Hue, Vietnam (X.C. Tran, L.V. An, N.T.P. Thao, P.K.C. Man);; Ewha Womans University, Seoul, South Korea (Y. Yun);; The Kyung Hee University, Seoul (S.-H. Kim);; The Jeju National University College of Medicine, Jeju, South Korea (J.R. Yoo, S.T. Heo, K.H. Lee);; Seoul National University College of Medicine, Seoul (N.-H. Cho)

**Keywords:** Severe fever with thrombocytopenia syndrome virus, Vietnam, SFTSV, viruses, ticks, vector-borne infections, phleboviruses, severe fever with thrombocytopenia syndrome, SFTS

## Abstract

Severe fever with thrombocytopenia syndrome (SFTS), a tickborne viral disease, has been identified in China, South Korea, and Japan since 2009. We found retrospective evidence of SFTS virus (SFTSV) infection in Vietnam, which suggests that SFTSV infections also occur in Vietnam, where the virus has not been known to be endemic.

Severe fever with thrombocytopenia syndrome virus (SFTSV) is a tickborne virus (genus *Phlebovirus*, family *Phenuiviridae*) that can cause a mild to severe febrile illness similar to hemorrhagic fever (*1*). Phleboviruses have been found in the Americas, Asia, Africa, and the Mediterranean region. For example, Heartland virus (HRTV), another tickborne phlebovirus, was identified in northwestern Missouri, USA, in 2009 (*2*). Malsoor virus, a new bat phlebovirus closely related to SFTSV and HRTV, was identified in western India, and a phlebovirus similar to SFTSV and HRTV was isolated from ticks in Australia (*3**,**4*).

Severe fever with thrombocytopenia syndrome (SFTS) illness was first confirmed in China in 2009. It was retrospectively identified in South Korea in 2010 and the western regions of Japan in 2013 (*1**,**5**,**6*). SFTS is characterized by acute high fever, thrombocytopenia, leukopenia, elevated serum hepatic enzymes, gastrointestinal symptoms, and multiorgan failure and has a death rate of 16.2%–30% (*1**,**6**,**7*). Atypical signs and symptoms and asymptomatic infections also have been identified (*5**,**8*). Most SFTSV infections occur through *Haemaphysalis longicornis* ticks, although SFTSV transmission can also occur through close contact with an infected patient (*8*).

To investigate evidence of SFTSV infections in Vietnam, we collected serum samples from 80 patients with acute febrile illnesses admitted to Hue University Hospital (Hue, Vietnam) during October 1, 2017–March 31, 2018. The Institutional Review Board of Hue University Hospital approved the study.

For the molecular diagnosis of SFTSV, we extracted RNA from stored patient serum using a QIAamp Viral RNA Mini Kit (QIAGEN, https://www.qiagen.com) and performed real-time reverse transcription PCR (rRT-PCR) to amplify the partial small (S) segment of the viral RNA from the stored serum and confirm SFTSV infection (*9*). rRT-PCR showed 2 positive results, from the stored serum of 2 patients with thrombocytopenia who had been seen at Hue University Hospital during 2017 and who had no history of travel to SFTSV-endemic countries, such as China, South Korea, and Japan. We also detected IgM in the serum of 1 of these patients (Appendix Table) (*8*).

On October 29, 2017, a 29-year-old woman (Hue 06-Vietnam-10-2017) was hospitalized at Hue University Hospital because of headache, vomiting, and gum bleeding. She lived in Hue City and was unaware of having received an insect bite. Her temperature was 38°C, and blood tests showed leukopenia (leukocyte count 1,900 cells/μL [reference 4,000–10,000 cells/μL]), thrombocytopenia (platelet count 125 × 10^3^/μL [reference 150–450 × 10^3^/μL]), and a low hematocrit level (34.3% [reference 36%–44%]). The patient fully recovered without other complications after 5 days.

On November 2, 2017, a 27-year-old man (Hue 13-Vietnam-11-2017) was hospitalized at Hue University Hospital because of headache and fatigue. He had had dengue fever at 8 years of age. Blood tests showed thrombocytopenia (platelet count 14 × 10^3^/μL), normal leukocyte count (7,410 cells/μL), mildly elevated aspartate aminotransferase (84 IU/L [reference 8–38 IU/L]), elevated alanine aminotransferase (98 IU/L [reference 4–44 IU/L]), and mildly elevated hematocrit (47.6% [reference 36%–44%]). He fully recovered without other complications after 7 days.

We sequenced rRT-PCR products from the stored serum samples using a BigDye Terminator Cycle Sequencing kit (Applied Biosystems, http://www.thermofisher.com). We performed phylogenetic analysis of the partial S segment sequences with MEGA6 (https://www.megasoftware.net) and constructed phylogenetic trees using the maximum-likelihood method, which confirmed SFTSV infection (Appendix Figure).

We confirmed 2 SFTSV infections in Hue in 2017 by amplifying the partial S segment of the viral RNA in stored serum from patients with thrombocytopenia; elevated levels of serum hepatic enzymes, including aspartate aminotransferase and alanine aminotransferase; and gastrointestinal symptoms, such as vomiting. The signs and symptoms were milder than the major signs and symptoms of SFTS, which has a high death rate.

*H. longicornis*, *Amblyomma testudinarium*, and *Ixodes nipponensis* ticks are vectors of SFTSV, and *A. testudinarium* has been found in Vietnam. Migratory birds are known to be long-distance carriers of virus-bearing ticks (*10*). Therefore, virus-bearing *A. testudinarium* ticks and migratory birds may play a role in dispersing SFTSV to Vietnam (*10*).

This study expands the understanding of the distribution of SFTSV in Southeast Asia and suggests that SFTSV may have a much wider global distribution than previously thought. The 2 patients reported here had relatively mild illness, and 1 did not have leukopenia. Therefore, further epidemiologic and clinical research is needed to clarify the epidemiology, geographic distribution, and transmission dynamics of SFTSV in Vietnam and other areas of Southeast Asia. This subject deserves further discussion and might warrant changes in the background description of the disease (*5**,**8*).

Appendix Additional data for endemic severe fever with thrombocytopenia syndrome, Vietnam.
