# Supplementary material for: Endemic Severe Fever with Thrombocytopenia Syndrome, Vietnam
Source: Emerg Infect Dis. 2019 May;25(5):1029–31. doi: 10.3201/eid2505.181463 (PMC6478219; doi:10.3201/eid2505.181463)
Supplement: Appendix — Additional data for endemic severe fever with thrombocytopenia syndrome, Vietnam. [file 18-1463-Techapp-s1.pdf]

Article DOI: https://doi.org/10.3201/eid2505.181463

Endemic Severe Fever with Thrombocytopenia Syndrome, Vietnam

Appendix

Appendix Table. Detection of severe fever with thrombocytopenia syndrome virus antibody, Vietnam

No	Patient	IgM-capture ELISA, optical density	IgG-capture ELISA, optical density
1	Hue 06-Vietnam-10–2017	+3.48	–1.69
2	Hue 13-Vietnam-11–2017	+/–1.05	–1.35
3	Positive control	1.73	2.60
4	Negative control	0.23	0.14

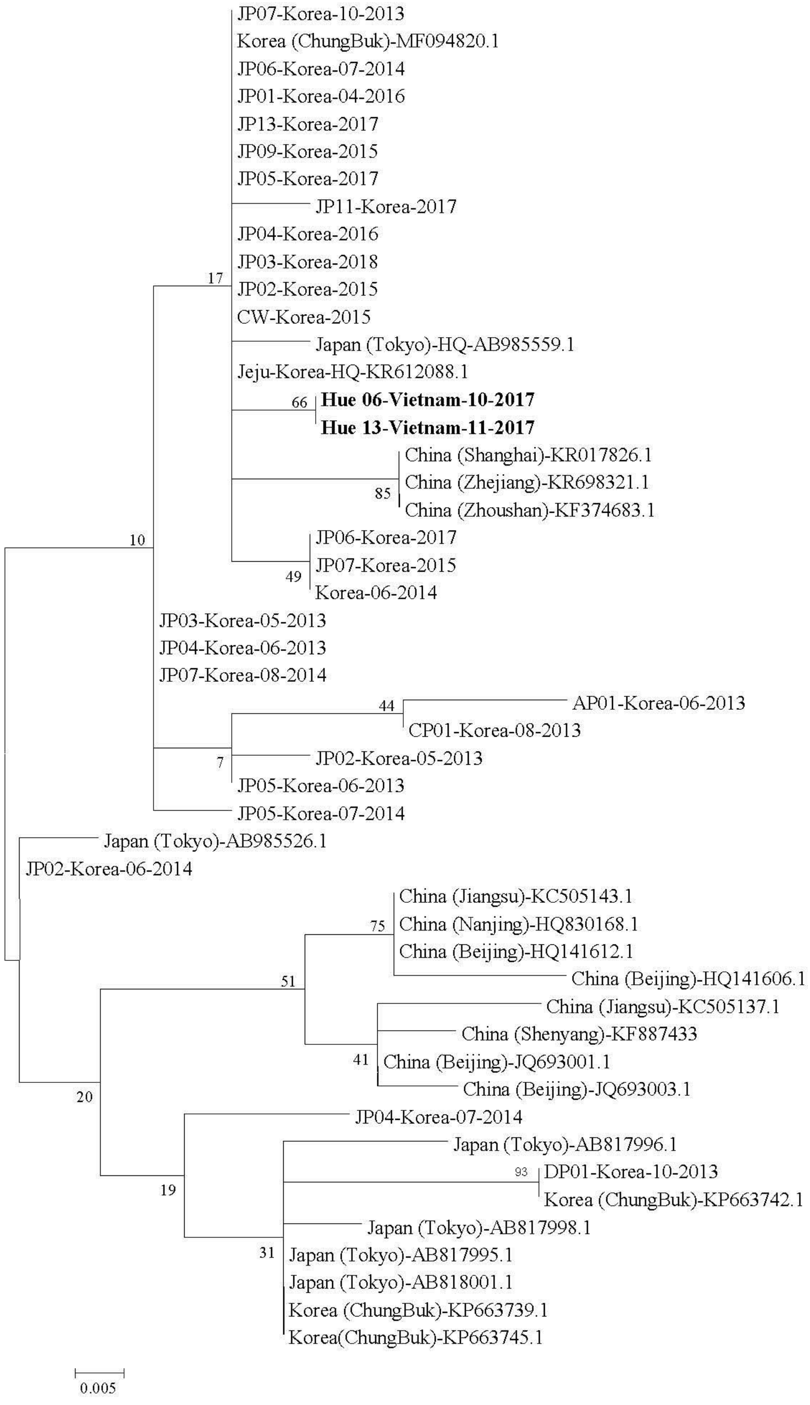

Appendix Figure. Phylogenetic tree constructed based on partial small (S) segment sequences of severe fever with thrombocytopenia syndrome virus. The tree was constructed using the maximum-likelihood method with MEGA 6. The partial S sequences from stored serum collected in Vietnam in 2017 and analyzed in this study are shown in bold (Hue-06-Vietnam-10–2017 and Hue-13-Vietnam-11–2017). The partial S sequence data of the viruses identified in China, South Korea, and Japan were obtained from NCBI/BLAST (http://blast.ncbi.nlm.nih.gov/Blast.cgi).
